# CXCR3 signaling promotes Delta One T cell recruitment and antitumor efficacy in colorectal cancer

**DOI:** 10.1136/jitc-2025-014668

**Published:** 2026-05-28

**Authors:** Mariana Carreira, Leandro Barros, Rafael M Cruz, Cristina Ferreira, Carla Costa, Carlos M Ferreira, Sofia Mensurado, Bruno Silva-Santos, Rafael Blanco-Domínguez

**Affiliations:** 1 GIMM, Lisbon, Portugal; 2 Hospital de Santa Maria, Lisbon, Portugal; 3 Universidade de Lisboa, Lisboa, Portugal

**Keywords:** Adoptive cell therapy - ACT, Colorectal Cancer, T cell

## Abstract

Immunotherapy has transformed cancer treatment, yet its efficacy in solid tumors, and particularly in colorectal cancer (CRC), remains limited by insufficient infiltration of endogenous or adoptively transferred immune cells. Our group developed Delta One T (DOT) cells, an allogeneic Vδ1-enriched γδ T-cell product with demonstrated efficacy in CRC models, but the critical mechanisms guiding their tumor homing are unclear. Here we show that DOT cells acquire a specific chemokine receptor profile during expansion to support tumor infiltration, characterized by prominent CXCR3 expression. Importantly, CXCR3 ligands (CXCL9–11) are expressed by CRC cell lines and primary tumors, and correlate with Vδ1 T cell infiltration and DOT-cell recruitment. CXCR3 signaling is critical for DOT-cell migration in vitro and in vivo, as pharmacologic blockade reduced tumor homing, whereas enhancing CXCR3 ligand expression increased DOT-cell infiltration and improved tumor control. These findings establish CXCR3 signaling as a key regulator of DOT-cell trafficking and a prime target to boost γδ T cell-based immunotherapies for CRC.

## Introduction

The emergence of immunotherapeutic approaches based on γδ T cells has positioned this unconventional lymphocyte subset as a promising candidate for “off-the-shelf” cellular therapies. γδ T cells combine varied tumor recognition mechanisms, potent antitumor activity, natural tissue homing properties, and lack of major histocompatibility complex restriction, features that enable broad tumor targeting with allogeneic suitability and reduced risk of graft-versus-host disease.^1^

Colorectal cancer (CRC) is the second leading cause of cancer mortality worldwide. Although immunotherapies have transformed outcomes in other cancers, their impact in CRC has been limited. The efficacy of immune checkpoint inhibitors (ICIs) is mostly restricted to 5–15% of CRC cases, explicitly those exhibiting DNA mismatch repair deficiency (MMR-d). These tumors accumulate large numbers of mutations, generating many neoantigens that elicit abundant αβ T cell infiltration and activation. However, most CRC cases are mismatch repair proficient (MMR-p), which lack sufficient neoantigens, and show minimal αβ T cell infiltrates, resulting in resistance to ICI.^2^ As in other solid cancers, adoptive cell therapies, including chimeric antigen receptor (CAR)-expressing T cells, also face major hurdles in CRC, including limited infiltration into the tumor core and exposure to an immunosuppressive tumor microenvironment (TME).

γδ T cells, and particularly their Vδ1 T cell receptor (TCR)-expressing subset, are enriched in epithelial tissues where they contribute to tissue surveillance, barrier integrity, and immune defense. In the context of CRC, Vδ1 T cells are frequently found within tumor lesions, where they constitute the main γδ T cell subset, and have been shown to exert cytotoxic activity against malignant epithelial cells with a limited degree of exhaustion.^3^ Moreover, their infiltration correlates with favorable clinical outcomes, underscoring their relevance as natural antitumor effectors in the gut.^4^ Leveraging these properties, our group developed Delta One T (DOT) cells, a clinical-grade Vδ1-biased γδ T cell product generated by expansion of peripheral blood cells from healthy donors.^5^ In addition to controlling hematological malignancies, DOT cells have demonstrated the ability to infiltrate and control xenografted CRC tumors in preclinical models.^6^ These findings suggest that Vδ1-enriched cell products could overcome limitations faced by conventional adoptive cell therapies in CRC. However, the mechanisms by which blood-derived DOT cells are recruited to CRC or other tumor sites remain poorly understood.

In fact, immune cell infiltration is a critical determinant of tumor control and patient outcome across solid tumors, including CRC. Chemokine gradients within the TME promote the recruitment of immune effectors, including natural killer (NK), αβ T, and γδ T cells. Among the chemokine axes implicated in tumor immunity, the CXCR3–CXCL9/10/11 pathway has been consistently important in directing effector cell trafficking and has been associated with improved prognosis in several cancers.^7^ CXCR3 is a G-protein coupled receptor that binds to three interferon (IFN)-inducible ligands: CXCL9 (MIG), CXCL10 (IP-10), and CXCL11 (I-TAC). However, the involvement of the CXCR3-CXCR3 ligand axis in mediating Vδ1/DOT cell trafficking to tumors remains unknown.

Here we investigated the chemokine receptor profile of DOT cells and its functional role in CRC infiltration. We identify CXCR3 as a key mediator of DOT-cell migration toward CRC tumors and show that enhancing engagement with CXCL9–11 ligands in the TME improves DOT-cell infiltration and antitumor activity. Our findings highlight the CXCR3 axis as a promising target to potentiate the therapeutic efficacy of Vδ1-enriched γδ T cell products in CRC.

## Materials and methods

### DOT-cell expansions

DOT cells were expanded as previously reported^5^ with slight modifications as described. Buffy coats from anonymous healthy donors were provided by the Instituto Português do Sangue e da Transplantação and used to generate DOT cells following institutional approval. Peripheral blood mononuclear cells (PBMCs) were isolated by Ficoll-Hypaque gradient centrifugation and subsequently depleted of αβ T cells by incubation with anti-TCR αβ-Biotin monoclonal antibody (Miltenyi Biotec) followed by anti-Biotin microbeads (Miltenyi Biotec). After magnetic separation using LS columns (Miltenyi Biotec), αβ-depleted PBMCs were resuspended in OpTmizer-CTS medium (Gibco) supplemented with 2.5% heat-inactivated human plasma Octaplas (Octapharma), 2 mmol/L L-glutamine (Thermo Fisher Scientific), 50 U/mL and 50 µg/mL of Penicillin/Streptomycin (Thermo Fisher Scientific), and 0.1% of heparin (Braun), from now on referred to as Complete-Optimizer. Cells were then cultured for 14 days in G-REX platforms (Wilson Wolf Manufacturing) at 37°C and 5% CO_2_. On day 0, anti-CD3 (clone OKT-3, 140 ηg/mL; BioLegend) and recombinant human cytokines, including IL-4 (100 ηg/mL), IFNγ (70 ηg/mL), IL-21 (7 ηg/mL), IL-1β (15 ηg/mL; all from PeproTech) were added to the medium. On day 7, cultures were supplemented with anti-CD3 (clone OKT-3, 1 µg/mL), rIL-21 (13 ηg/mL), and rIL-15 (70 ηg/mL). On day 11, cells received fresh medium containing anti-CD3 (1 µg/mL) and rIL-15 (100 ηg/mL). On day 14, DOT cells were harvested, phenotyped and cryopreserved in CryoStor CS10 (Merck), and stored in liquid nitrogen until use. Only DOT cell expansions reaching ≥65% Vδ1^+^ T cells were used for experiments.

To evaluate CXCR3 expression across Vδ1^+^ differentiation subsets, αβ-depleted cells were sorted into CXCR3^−^ Vδ1^+^, CXCR3^+^ Vδ1^+^, and Vδ1^−^ populations. For DOT cell expansion, 10³ CXCR3^−^ Vδ1^+^ or CXCR3^+^ Vδ1^+^ cells were cultured in 96-well plates in the presence of 49×10³ autologous Vδ1^−^ feeder cells (5×10^4^ cells/well in total). After sorting, Vδ1^+^ cells were expanded using an adapted version of the previously described DOT-cell protocol. Cells were cultured in Complete-Optimizer supplemented with anti-CD3 (1 µg/mL) and the recombinant human cytokines IL-4 (100 ηg/mL), IFNγ (70 ηg/mL), IL-21 (7 ηg/mL) and IL-1β (15 ηg/mL). On day 7, fresh medium containing anti-CD3 (2 µg/mL), rIL-21 (13 ηg/mL), and rIL-15 (70 ηg/mL) was added to the wells. On day 11, cells from each well were resuspended and distributed into six replicate wells. Fresh medium supplemented with anti-CD3 (2 µg/mL) and rIL-15 (100 ηg/mL) was then added to all wells. DOT cells were harvested and phenotyped on day 14.

### Cancer cell lines

The human colon cancer cell lines HCT-116 and SW620 were purchased from the American Type Culture Collection (ATCC, references #CCL-247 and #CCL-227), respectively). SW620 Luc^+/+^ GFP^high^ cells were generated by transducing SW620 cells with the lentivirus CMV-Luciferase (firefly)-2A-GFP (#LVP020, Amsbio). Both cell lines were cultured in Roswell Park Memorial Institute (RPMI) 1640 medium (Gibco) supplemented with 10% fetal bovine serum (FBS, Gibco), 1% Penicillin/Streptomycin (Gibco), and 0.2% MycoZap (Lonza). Cells were grown in T75 flasks (75 cm^2^; Nunc EasYFlask; Thermo Scientific) with 15 mL of medium and maintained in a humidified incubator at 37°C and 5% CO_2_. Assays were performed with cells reaching 60–80% optical confluency. At this point, the medium was removed, and cells were washed with 8 mL of phosphate-buffered saline (PBS). Detachment of cells was achieved by incubating them with 2 mL of TrypLe-Express (1X, phenol red, Gibco) for 5 min at 37°C.

### Orthotopic CRC xenograft model

hIL15-NOG female mice (NOD.Cg-*Prkdc*^scid^ *Il2rg*^tm1Sug^ Tg(CMV-IL-2/IL-15)1-1Jic/JicTac) were purchased from Taconic Biosciences. Animals aged 7–20 weeks were used for experiments and housed under specific pathogen-free conditions at GIMM Rodent Platform. The orthotopic CRC xenograft model was generated as previously described.^6^ In brief, mice were anesthetized with 3% isoflurane in oxygen delivered via a nose cone and 10^5^ SW620 Luc^+/+^ GFP^high^ cells, suspended in 20 µL of PBS, were carefully inoculated into the exposed cecal serosa, between the epithelial layers of the cecal wall. After repositioning the cecum, the peritoneum was sutured, and mice received a subcutaneous administration of 100 µL of buprenorphine (0.3 mg/mL) for analgesia.

Tumor growth was assessed in all animals using the IVIS Lumina Fluorescence and Bioluminescence Imaging System (Caliper, LifeSciences). Mice were anesthetized via intraperitoneal injection of 200 µL of anesthetic solution (7.5 mg/mL ketamine and 0.1 mg/mL medetomidine in water), followed by an intraperitoneal injection of 200 µL of 15 mg/mL of XenoLight D-Luciferin—K+ Salt Bioluminescent Substrate (Revvity). After 7 min, luminescence was measured with 30 and 60 s of exposure, and the highest value was considered for analysis. At last, 200 µL of 0.1 mg/mL Antisedan (Esteve) was administered intraperitoneally for recovery. Luminescence was analyzed by Living Image V.3.0 Software, and tumor size was evaluated as photons released per second. All animal experiments were approved by the Animal Welfare Body at GIMM and the national competent authority (DGAV) under the license number 0421/000/000/2023.

### In vivo treatments

Mice were assigned randomly across experimental groups, and all treatment protocols were initiated following tumor confirmation by luminescence assays. To assess DOT-cell infiltration into CRC tumors, mice received weekly intravenous injection of 10^7^ DOT cells, suspended in 100 µL of Optimizer (Gibco), for 3 weeks.

For CXCR3-loss-of-function assays, mice were treated with the CXCR3 antagonist AMG487 3 weeks after tumor implantation, following either a systemic or targeted strategy. In the systemic approach, mice received a single intravenous injection of DOT cells followed by daily intraperitoneal administration of the CXCR3 antagonist AMG487 (5 mg/kg; MedChem) for 1 week. The antagonist was prepared in 100 µL of vehicle consisting of 10% dimethyl sulfoxide and 90% corn oil (MedChem). In the targeted approach, DOT cells were pretreated in vitro with AMG487 (1 µM; MedChem Express) and recombinant human IL-15 (100 ηg/mL) for 1 day before infusion. Then, 10^7^ DOT cells were intravenously injected into tumor-bearing mice. Mice were sacrificed 1 week after injection to assess DOT cell infiltration into tumors.

For in vivo AC484 treatment, the drug was formulated in ethanol, PEG400, and Phosal-50PG at a 10:30:60 ratio (vehicle). Mice received AC484 (20 mg/kg) 8 or the vehicle by oral gavage daily during the first 3 days of treatment, starting on day 6 after tumor inoculation and then every other day for 18 days. In parallel, 10^7^ DOT cells were intravenously injected on days 7, 14, and 21.

### Primary tumor samples from patients with CRC

Colorectal tumor specimens were collected from non-treated patients via colonoscopy between January 2023 and November 2025 at Hospital Santa Maria (Lisbon, Portugal). Following surgical excision, tumor specimens were maintained in DMEM (Gibco) supplemented with 5% Penicillin/Streptomycin (Gibco), 5% Fungizone/Amphotericin B (Gibco), 0.2% Gentamicin (Gibco) and 0.1% Metronidazole (Duchefa Biochemie). Within the first 3 hours from collection, tumor specimens were cut into 1–2 mm^3^ fragments and cryopreserved in CryoStor CS10 cell cryopreservation media (Merck) in liquid nitrogen until use. For experimental assays, biopsies were processed following the published protocol.^9^ Cryopreserved tumor specimens were thawed prior to processing while fresh specimens were processed directly. For both fresh and thawed cryopreserved samples the medium was removed, and the fragments were transferred to a Falcon tube with 1 mL of DMEM supplemented with 0.1 mg/mL of DNase I (Roche) and 0.25 U/mL Liberase (Roche). Tumor specimens were mechanically chopped into smaller fragments to facilitate the enzymatic action and incubated with an additional 4 mL of digestion medium for 20 min at 37°C with agitation at 100 rpm. Following enzymatic digestion, cell suspensions were passed through a 100 µM filter, then washed and resuspended for further use in RPMI-1640 (Gibco) supplemented with 10% FBS (Gibco), 1% CTS Glutamax (Gibco), 1% MEM Non-Essential Amino Acids Solution (Gibco) and 0.2% Mycozap (Lonza), from now referred to as Complete-RPMI.

### In vitro chemotaxis assays

DOT cell migration was evaluated using 96-well Transwell plates (5 µM pore size, Corning). DOT cells were thawed and rested overnight with rIL-15 (100 ηg/mL; PeproTech) prior to the assay. On the day of the experiment, 2×10^5^ DOT cells were resuspended in 100 µL of Complete-RPMI and carefully loaded onto the Transwell inserts.

To evaluate DOT cell responsiveness to chemotactic cues, recombinant chemokines (CCL2, CCL5, CCL17, CCL20, CCL22, CXCL9, CXCL10, CXCL12, and CXCL16, all from PreproTech) were added to the lower wells in Complete-RPMI, at 1, 10, and 100 ηg/mL, immediately before the assay.

Additionally, CTV-stained SW620, HCT-116 cells, and single-cell suspensions of cryopreserved patient-derived CRC specimens, were seeded at increasing densities in the lower chambers in Complete-RPMI and incubated overnight at 37°C.

For CXCR3 loss-of-function assays, DOT cells were either treated with the CXCR3 antagonist AMG487 (1 µM, MedChem Express) during the overnight culture or preincubated with an anti-human CXCR3 monoclonal antibody (20 µg/mL, Clone 49801, R&D Systems) for 15 min at 4°C, immediately before the migration assay. To establish a chemotactic gradient, 8×10^5^ tumor cells were seeded in the lower chambers and incubated overnight to allow ligand secretion. Recombinant human CXCL10 (10 ηg/mL; BioLegend) was used as a positive control. As a complementary approach, SW620 cells were stimulated with rIFNγ (10 ηg/mL) and cultured for 3 days at 37°C and 5% CO_2_ to generate chemokine-enriched supernatants. These supernatants were then placed in the lower chambers of Transwell plates, either alone or in combination with neutralizing antibodies against human CXCL9 (20 µg/mL, clone 49106, R&D Systems), CXCL10 (20 µg/mL, clone 33036, R&D Systems), or CXCL11 (20 µg/mL, clone 87328, R&D Systems) for 1 hour at 37°C before initiating the migration assay. For gain-of-function experiments, tumor cells cultured in the lower wells were supplemented with ABBV-CLS-484 (AC484; 10 µM; MedChemExpress) along with rIFNγ and incubated overnight at 37°C.

Single-cell suspensions of fresh patient-derived CRC specimens were used as chemoattractants. After processing, CD45^−^ cells were isolated from the initial sample through MACs separation (Miltenyi Biotec) and seeded in the lower chambers in complete RPMI. Cells were cultured in the presence or absence of AC484 (10 µM; MedChemExpress) and IFNγ (100 ηg/mL), and incubated overnight at 37°C.

In all experiments, DOT cells loaded into the upper chambers of the Transwell were incubated for 6 hours at 37°C. Migrated DOT cells were then collected from the lower chambers, quantified and stained with fluorochrome-conjugated antibodies against surface markers. The chemotaxis index was calculated as the ratio of the number of DOT cells that migrated in each condition to the number that migrated in control wells without tumor cells.

### Multiplex analysis of chemokines

SW620 and HCT-116 cells (4×10⁶ cells/mL) were cultured for 3 days in the presence or absence of rIFNγ (10 ηg/mL). Culture supernatants were collected, snap-frozen for preservation, and chemokine levels were analyzed using the Human Cytokine/Chemokine 96-Plex Discovery Assay (HD96, Eve Technologies Corporation). This analysis was performed using the Luminex xMAP technology, a multiplexed bead-based platform that employs internally color-coded beads conjugated with specific capture antibodies. This system allows for the simultaneous detection and quantification of ninety-six human cytokines, chemokines, and growth factors.

### Real-time quantitative PCR

Total RNA was extracted with the High Pure RNA Isolation Kit (Roche) and reverse-transcribed into cDNA (Promega). Real-time quantitative PCR (RT-qPCR) was performed on a ViiA 7 Real-Time PCR System (Applied Biosystems) using SYBR Green Reagents (Thermo Fisher Scientific). Primer sequences are listed in online supplemental table 1. Messenger RNA (mRNA) levels were determined using the 2^−ΔΔCT^ method and normalized to the expression of the housekeeping gene β-actin.

10.1136/jitc-2025-014668.supp2 Supplementary data

### ELISA

Supernatants from SW620 and HCT-116 cells cultured for 3 days at 37°C, with or without rIFNγ (100 ηg/mL), were collected and stored at −80°C until use. In parallel, supernatants from the migration assays performed with CD45^−^ cells isolated from fresh CRC specimens were also collected and stored under the same conditions. CXCL10 levels were quantified using the Human IP-10 (CXCL10) Standard TMB ELISA Development Kit (PeproTech), following the manufacturer’s instructions.

### Organ processing for in vivo DOT-cell analyses

Animals were sacrificed by CO_2_ narcosis 4 weeks after tumor inoculation, and tumors, blood, and spleens were harvested. Tumors were cut into small pieces and processed in 1 mL of IMDM (Gibco) supplemented with 20% FBS, 1% Penicillin/Streptomycin, 1% Amphotericin B (Gibco), and 0,1% of 50 mg/mL Gentamicin (Gibco), from now referred to as Complete-IMDM. To ensure enzymatic digestion, 0.05 mg/mL collagenase IV (Roche) and 1 mg/mL DNAse I (Roche) were added to the eppendorfs with tumor cells. Samples were incubated for 30 min at 37°C with agitation at 900 rpm. The resulting cell suspensions were filtered through a 70 µM cell strainer using a syringe plunger, then washed and resuspended in Complete-IMDM for further flow cytometry analysis. Concurrently, spleens were mashed and filtered through a 70 µM cell strainer. Single-cell suspensions from blood and spleen were treated with red blood cell lysis buffer (BioLegend), washed, and resuspended in PBS for subsequent flow cytometry staining.

### Flow cytometry

Single-cell suspensions were incubated in PBS with fluorochrome-conjugated monoclonal antibodies against surface markers and viability dyes for 20 min at 4°C in the dark. To prevent non-specific binding, anti-mouse CD16/CD32 (Invitrogen, 14–0161-86) and/or anti-human Fc receptor (Invitrogen, 14–9161-73) antibodies were included during surface staining. After incubation, samples were washed and resuspended in PBS.

For intracellular marker evaluation, cells were prestimulated with 0.5% of Brefeldin A (Merck) and 0.1% of Monensin (Invitrogen) for 3 hours at 37°C to inhibit cytokine secretion. Surface staining was then performed as described above, followed by fixation and permeabilization using the Foxp3 Staining Buffer Set (Invitrogen), according to the manufacturer’s instructions. In brief, cells were fixed for 30 min at 4°C, washed with permeabilization buffer, and subsequently stained with fluorophore-conjugated antibodies targeting intracellular molecules for 1 hour at 4°C. At last, cells were washed and resuspended in PBS for flow cytometry acquisition.

Flow cytometry acquisition was performed on BD FACSymphony A5 SE (BD Biosciences) or Cytek Aurora (Cytek Biosciences). Data were analyzed with FlowJo software (Tree Star).

All antibodies and dyes used for flow cytometry are listed on the online supplemental table 2.

10.1136/jitc-2025-014668.supp3 Supplementary data

### TCGA gene expression

Gene expression data were retrieved from The Cancer Genome Atlas (TCGA) for colon adenocarcinoma and rectum adenocarcinoma cohorts. Expression values were normalized as fragments per kilobase of transcript per million mapped reads (FPKM) to allow comparison across datasets.

### Statistical analysis

Data normality was assessed using the Shapiro-Wilk test. For normally distributed data, two-group comparisons were performed using an unpaired Student’s t-test, or a paired t-test for matched samples. Comparisons involving more than two groups were analyzed by one-way analysis of variance (ANOVA) followed by either Dunnett’s or Tukey’s multiple-comparisons test. Dunnett’s test was used when comparing multiple groups to a single control, whereas Tukey’s test was applied for comparisons between all groups. For non-normally distributed data, the Mann-Whitney U test was used for two-group comparisons, and the Kruskal-Wallis test followed by Dunn’s multiple-comparisons test was used for comparisons among multiple groups. When two independent variables were analyzed, a two-way ANOVA followed by Šidák’s multiple-comparisons post hoc test was applied. Spearman’s rank correlation coefficient was used to assess associations between continuous variables. All data analyses were performed using GraphPad Prism V.8 Software and differences were considered statistically significant for p values <0.05. All p values are presented in the figures.

## Results and discussion

### DOT cells migrate toward CRC tumors via CXCR3

**Figure 1:**
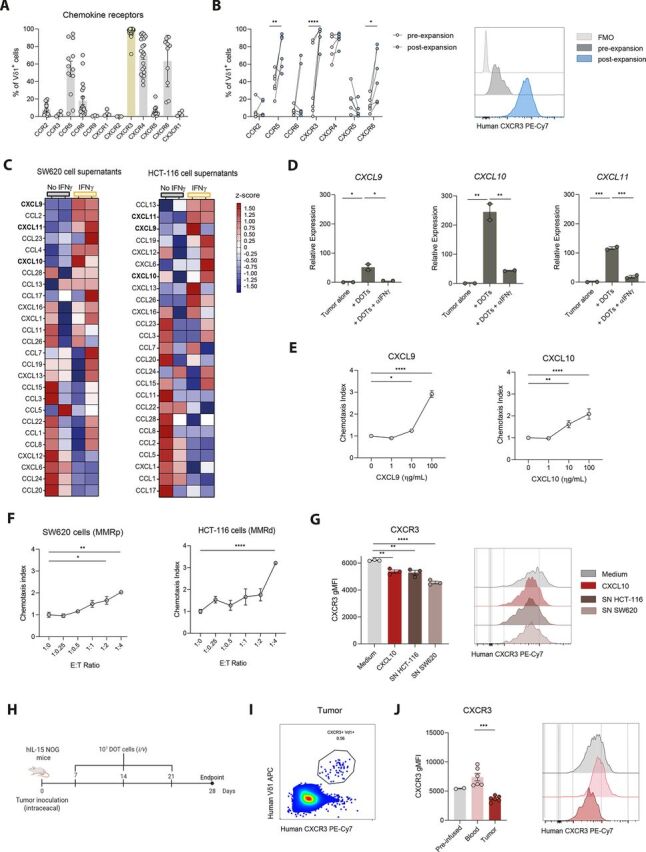
DOT-cell expansion protocol induces a tumor-homing chemokine receptor repertoire with dominant CXCR3 expression. (A) Chemokine receptor repertoire of different individual DOT donors analyzed following expansion (n=3–29 DOT donors), represented as means±SEM. (B) Expression of chemokine receptors and representative histogram of CXCR3 expression in Vδ1^+^ γδ T cells before and after DOT-cell expansion. Lines connect paired data points from matched donors (n=6 donors). Data were analyzed by a two-way ANOVA with Šidák’s multiple-comparisons test. (C) Heatmaps showing chemokine expression in SW620 and HCT-116 cancer cell lines following stimulation with rIFNγ (10 ng/mL). Data were standardized by Z-scores, calculated using the mean and SD for each row. Red and blue gradients indicate deviations above and below the mean, respectively. Chemokines are ranked by linear fold-change relative to unstimulated controls and CXCR3 ligands are highlighted in bold. (D) Relative mRNA expression of CXCR3 ligands by SW620 cells after 24 hours co-culture with freshly thawed DOT cells, with or without 10 µg/mL of neutralizing anti-IFNγ antibody (n=2 technical replicates), measured by qPCR. Data are representative of two independent experiments with similar trends. (E) Chemotaxis index of DOT cells after a 6-hour Transwell migration assay, calculated as the ratio of migrated DOT cells present in the lower wells versus the control (medium without chemoattractant). rCXCL9 and rCXCL10 were added to the lower wells at varying concentrations. One representative experiment out of three independent experiments, represented as mean±SEM. Data were analyzed by one-way ANOVA with Dunnett’s multiple comparisons test. (F) Chemotaxis index of DOT cells in the presence of SW620 or HCT-116 cells, seeded in the lower wells at increasing concentrations. Migration assays with cell lines are representative experiments (n=2–3 technical replicates). Data were analyzed by one-way ANOVA with Dunnett’s multiple comparisons test and are presented as mean±SEM. (G) gMFI of CXCR3 in Vδ1^+^ γδ T cells and representative histograms following a 6-hour migration assay with rCXCL10 or SNs from HCT-116 or SW620 cancer cell lines. Representative experiment with three technical replicates. Data are presented as means±SEM and were analyzed by a one-way ANOVA with Tukey’s multiple-comparisons test. (H) Schematic representation of the experimental approach to assess DOT-cell infiltration into orthotopic CRC tumors. 10 million DOT cells were injected intravenously once a week into SW620 xenograft mice. (I) Representative flow cytometry density plot showing the percentage of CXCR3^+^ Vδ1^+^ γδ T cells (gated on alive cells) present in the tumor of mice. (J) gMFI and representative histograms of CXCR3 expression on DOT cells before infusion into mice (n=2 technical replicates) and in blood and tumor samples (n=6 mice) 2 weeks after infusion. Data are presented as means±SEM and were analyzed by a one-way ANOVA with Tukey’s multiple-comparisons test. *p< 0.05, **p<0.01, ***p<0.001, ****p< 0.0001. ANOVA, analysis of variance; CRC, colorectal cancer; DOT, Delta One T; E:T Ratio, Effector-to-Target ratio; FMO, Fluorescence Minus One; gMFI, geometric mean fluorescence intensity; IFNγ, interferon-gamma; i/v, intravenous; MMRd, mismatch repair deficiency; MMRp, mismatch repair proficient; mRNA, messenger RNA; qPCR, quantitative PCR; SN, supernatant.

To investigate the mechanisms underlying DOT-cell trafficking to CRC, we characterized their chemokine receptor repertoire. We found that DOT cells express a repertoire consistent with gut and tissue tropism, with moderate expression of CCR2, CCR6, and CXCR5, and prominent expression of CCR5, CXCR3, CXCR4, and CXCR6. Among them, CXCR3 emerged as the most consistently and highly expressed receptor across donors, being expressed by virtually all DOT cells (figure 1A). To test if these receptors were induced by the DOT-cell expansion protocol,^5^ we analyzed their expression on Vδ1 T cells isolated from PBMCs of healthy donors before and after DOT-cell expansion. While the expression of most receptors was minimally affected, CXCR3 (and to a lesser extent CCR5 and CXCR6) was markedly upregulated following the DOT-cell expansion protocol (figure 1B and online supplemental figure S1A).

10.1136/jitc-2025-014668.supp1 Supplementary data

Considering this phenotype, we questioned whether the dominant CXCR3 expression on DOT cells was due to preferential expansion of CXCR3^+^ Vδ1 T-cells or to acquisition of receptor expression by CXCR3^−^ Vδ1 T-cells during the protocol. Phenotypic characterization of circulating Vδ1^+^ T cells before expansion confirmed that naïve Vδ1^+^ T cells (CD45RA^+^ CD27^+^) expressed higher CXCR3 levels than their effector counterparts (online supplemental figure S1B,C), aligning with previous literature.^10^ Importantly, sorted PBMCs-derived CXCR3^+^ Vδ1^+^ T cells, containing a higher proportion of naïve cells (online supplemental figure S1D), achieved higher fold-expansion than CXCR3^−^ counterparts under the DOT-cell protocol (online supplemental figure S1E), consistent with naïve cells being more prone to expand. Nevertheless, CXCR3^−^ cells also uniformly acquired prominent CXCR3 expression after the DOT-protocol (online supplemental figure S1F), indicating that the elevated CXCR3 expression observed in DOT cells arises from a combination of preferential expansion of naïve Vδ1^+^ T cells and activation-induced receptor upregulation.

CRC cells secrete a wide range of factors that shape immune cell recruitment, tumor progression, and treatment response. Secretome profiling of human CRC cell lines (MMR-p SW620 and MMR-d HCT-116) confirmed that their supernatants contain all three CXCR3 ligands CXCL9, CXCL10, and CXCL11, alongside a broad spectrum of additional chemokines (figure 1C and online supplemental figure S2A). Since CXCL9-11 are IFN-inducible ligands, we compared their secretion with and without IFNγ stimulation. IFNγ markedly increased the levels of all three ligands, indicating that exposure to IFNγ producers and/or modulation of the IFNγ pathway could further enhance their production (figure 1C and online supplemental figure S2A). Effector immune cells are the primary source of IFNγ in the TME. Through activation of the Janus kinase (JAK)-signal transducer and activator of transcription (STAT) pathway, and induction of IFN-responsive genes, high IFNγ levels in the TME might prime CRC tumors to produce CXCR3 ligands. Accordingly, the presence of DOT cells, known to produce abundant IFNγ,^5^ increased expression of these ligands in tumor cells, and this was mediated by IFNγ, as it was abolished upon its neutralization (figure 1D).

Through Transwell chemotaxis assays, we observed that DOT cells migrated in a dose-dependent manner toward recombinant CXCL9 and CXCL10, confirming functional CXCR3-dependent chemotaxis (figure 1E). Migration toward other chemokines implicated in immune cell trafficking and tumor infiltration,^11–14^ whose receptors were also expressed—although at lower levels—by DOT cells, was comparatively weaker (online supplemental figure S2A). Although additional mechanisms may contribute to DOT-cell recruitment to CRC tumors, exposure to equivalent ligand concentration ranges, within those detected in CRC cell supernatants (online supplemental figure S2B), elicited a stronger migratory response toward CXCR3 ligands, positioning CXCR3 as a dominant driver of DOT-cell trafficking. Consistent with the expression of CXCR3 ligands by CRC cells, DOT cells showed active migration toward both SW620 and HCT116 CRC cell lines (figure 1F). Downregulation of CXCR3 on exposure to chemokine-rich supernatants from CRC cells, mimicked exposure to recombinant CXCL10 and evidenced receptor engagement (figure 1G).

To explore this mechanism in vivo, we employed an orthotopic (intracecum) CRC xenograft murine model, previously established in our laboratory, and reported to efficiently recruit adoptively transferred DOT cells.^6^ Following tumor detection by bioluminescence, DOT cells were infused weekly (figure 1H). Tumor-infiltrating DOT cells displayed reduced CXCR3 levels compared with circulating or preinfused DOT cells (figure 1I–J), consistent with receptor engagement in vivo. These data collectively support that CXCR3 is a key determinant of DOT-cell migration in preclinical CRC models.

### CXCR3 ligand expression in tumors from patients with CRC associates with δ1 T cell recruitment

**Figure 2:**
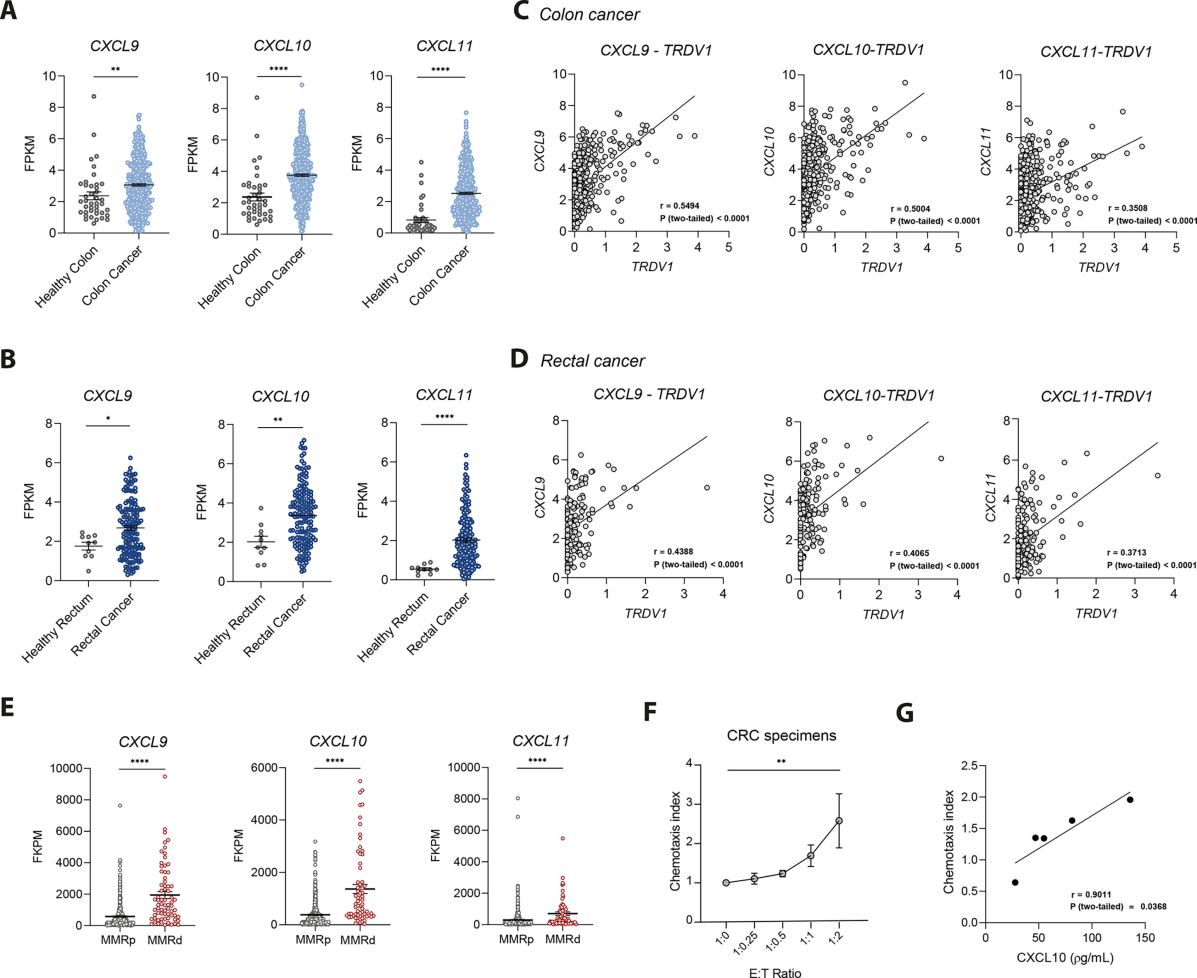
CXCR3 ligand expression in primary CRC correlates with endogenous Vδ1 T cell infiltration. (A) Analysis of CXCR3 ligands mRNA expression in healthy colon tissue (n=41) and primary colon cancer (n=453) from TCGA repository. Data were analyzed by Mann-Whitney U test. (B) Analysis of CXCR3 ligands expression in healthy rectum (n=10) and primary rectal cancer (n=163) from TCGA. Data were analyzed by unpaired t-test, in the case of *CXCL10* (normal distribution), and Mann-Whitney U test, for *CXCL9* and *CXCL11* (non-normal distributions). (C) Correlation of CXCR3 ligands and *TRDV1* mRNA expression in primary samples of colon cancer (n=453). (D) Correlation of CXCR3 ligands and *TRDV1* mRNA expression in primary samples of rectal cancer (n=163). Spearman’s correlation coefficients (r) and p values are shown. (E) Expression of *CXCL9, CXCL10,* and *CXCL11* in primary samples from MMR-p (n=468) and MMR-d (n=70) CRC tumors. Data were analyzed by the Mann-Whitney U test. (F) Chemotaxis index of DOT cells in response to CD45-depleted cells isolated from cryopreserved primary CRC specimens, seeded in the lower wells at increasing concentrations. Pool of three independent experiments (n=5 CRC specimens from different donors). Data were analyzed by one-way ANOVA with Dunnett’s multiple comparisons test and are presented as mean±SEM. (G) Correlation between the chemotaxis index of DOT cells in the presence of CD45^−^ cells isolated from fresh primary CRC specimens and CXCL10 levels measured in the corresponding CD45^−^-cell supernatants. Data represent a pool of three independent experiments (n=5 donors). Data in panels A–E were obtained from the TCGA repository and quantified as FPKM. *p< 0.05, **p<0.01, ****p< 0.0001. ANOVA, analysis of variance; CRC, colorectal cancer; DOT, Delta One T; E:T Ratio, Effector-to-Target ratio; FPKM, fragments per kilobase of transcript per million mapped reads; MMRd, mismatch repair deficiency; MMRp, mismatch repair proficient; mRNA, messenger RNA; TCGA, The Cancer Genome Atlas.

To investigate whether the CXCR3 axis may govern tumor infiltration of endogenous Vδ1^+^ T cells in patients with CRC, we analyzed publicly available datasets from TCGA. *CXCL9*, *CXCL10,* and *CXCL11* were overexpressed in both primary colon and rectal cancers when compared with the respective healthy tissues (figure 2A–B). Importantly, expression of CXCR3 ligand-encoding genes positively correlated with *TRDV1* (the gene encoding the TCR Vδ1 and a proxy of Vδ1^+^ T cells) in primary samples from patients with colon or rectal cancer (figure 2C–D). Although an association between CXCR3 ligand expression and endogenous Vδ1^+^ T cell infiltration is inferred, these TCGA data are derived from bulk RNA sequencing. Spatially resolved analyses would be informative to confirm colocalization within the tumor microenvironment.

Given that MMR-d CRC tumors are typically associated with increased immune infiltration, whereas MMR-p CRC tumors are generally immune-cold,^15^ we compared chemokine expression between these two subtypes. MMR-d tumors displayed significantly higher expression of *CXCL9*, *CXCL10*, and *CXCL11* compared with MMR-p tumors (figure 2E), while interestingly no differences were detected for other studied chemokine ligands (online supplemental figure S2C). These data suggest that the CXCR3–CXCL9/10/11 axis may promote the recruitment of endogenous Vδ1^+^ T cells to tumors from patients with CRC, similarly to what we observed for DOT cells in CRC xenografted mice.

Additionally, to test whether DOT cells migrate toward patient-derived tumors using the same mechanism, we performed chemotaxis assays using cell suspensions from primary CRC specimens. To avoid intrinsic variability of immune cell infiltration into the specimens that could interfere with CXCR3 ligand levels (through either direct ligand production or IFNγ secretion), we depleted CD45^+^ cells from the primary CRC cell suspensions. In line with our findings with CRC cell lines, we observed that DOT cells migrated toward primary CRC specimens (figure 2F) and this migration strongly correlated with CXCL10 secretion (figure 2G). These data indicate that non-immune cells are a relevant source of CXCL10 in CRC and further support that CXCR3 ligands are key mediators of DOT-cell migration toward human CRC tumors. Collectively, our results underscore the role of CXCR3 ligands in promoting the recruitment of both endogenous Vδ1^+^ T cells and infused DOT cells into CRC tumors, leading us to explore the therapeutic potential of modulating this axis to enhance DOT-cell efficacy in preclinical CRC models.

### CXCR3 inhibition restricts DOT-cell migration toward CRC

**Figure 3:**
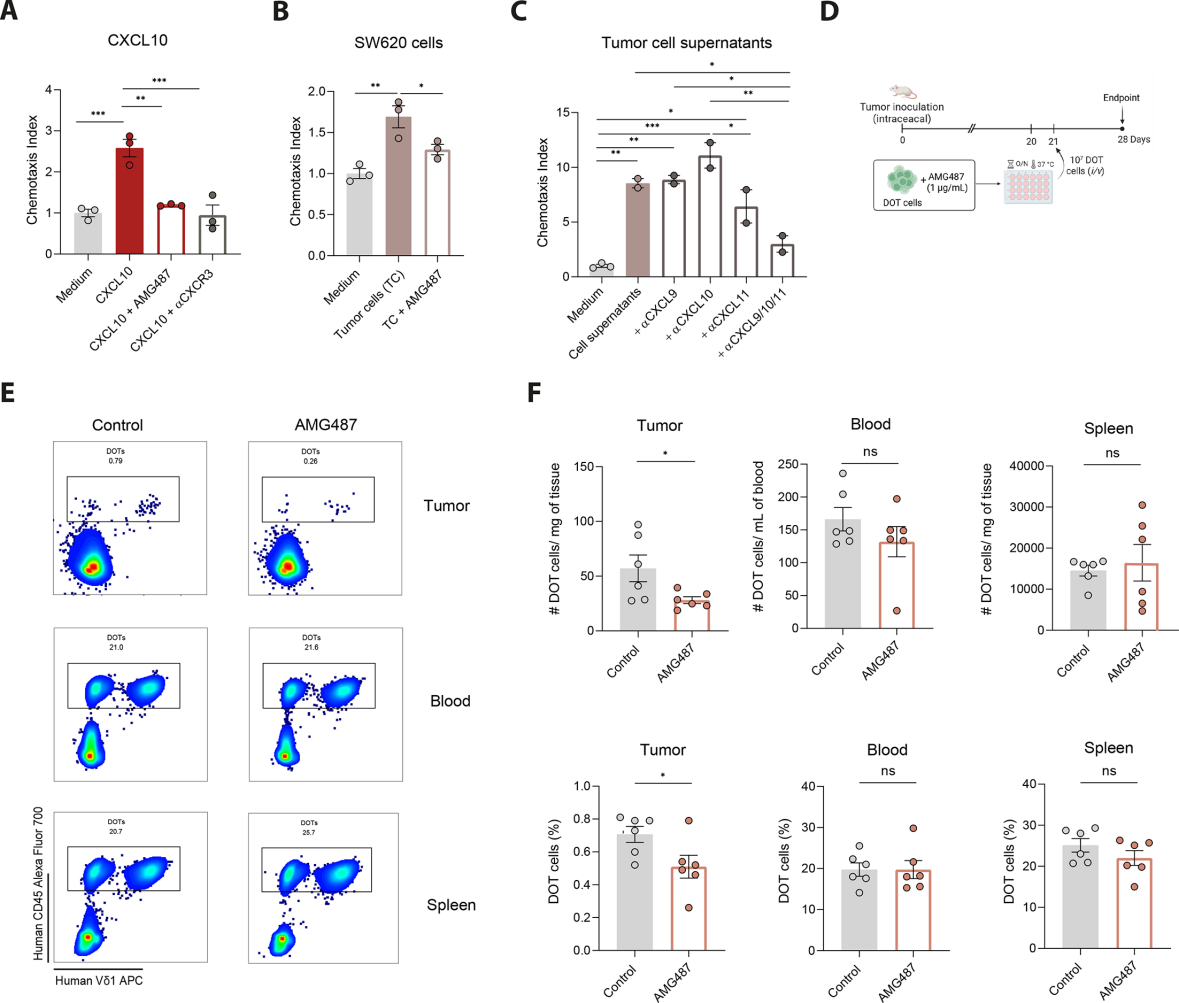
CXCR3 inhibition impairs DOT-cell migration in vitro and in vivo. (A) Chemotaxis index of DOT cells toward rCXCL10 (10 ηg/mL), with or without CXCR3 inhibition using a CXCR3 antagonist (AMG487, 1 µM) or a monoclonal antibody (αCXCR3, 20 µg/mL). Data represented as mean±SEM from one representative experiment out of four independent experiments and analyzed by one-way ANOVA with Holm-Šidák multiple comparison test. (B) Migration index of DOT cells, treated or not with AMG487 (1 µM), in the presence of 8×10^5^ SW620 cells incubated overnight. Data represented as mean±SEM from one representative experiment out of two independent experiments and analyzed by one-way ANOVA with Holm-Šidák multiple comparison test. (C) Chemotaxis index of DOT cells in response to chemokine-enriched supernatants from SW620 cells, with or without blocking antibodies against CXCL9, CXCL10, and/or CXCL11. One representative experiment out of two independent experiments, represented as mean±SEM. In A–C, medium without chemoattractant served as the control. (D) Experimental layout to assess the impact of CXCR3 inhibition on DOT cell infiltration into CRC tumors in vivo. 3 weeks post-tumor implantation, 10 million DOT cells, precultured with 1 µM of AMG487 for 24 hours, were injected intravenously into tumor-bearing mice. (E) Representative flow cytometry density plots showing the percentages of DOT cells (gated on alive cells) in tumor, blood, and spleen from AMG487-treated and untreated animals (n=6 mice per group). (F) Number of DOT cells (hCD45^+^ cells) normalized per mg of tissue or per mL of blood (upper panels) and total percentages of DOT cells (bottom panels) in the two experimental groups.In E–F, data are presented as mean±SEM and were analyzed using unpaired t-tests for normally distributed data or Mann-Whitney U tests for non-normally distributed data.*p< 0.05, **p<0.01, ***p<0.001. ANOVA, analysis of variance; CRC, colorectal cancer; DOT, Delta One T; IFNγ, interferon-gamma; i/v, intravenous; ns, not significant.

To formally demonstrate that CXCR3 mediates DOT-cell migration toward CRC, we performed loss-of-function assays using the clinically tested CXCR3 antagonist AMG487, which competitively inhibits ligand-receptor binding.^16^ As expected, AMG487 completely abrogated DOT-cell Transwell migration in response to CXCL10, resembling anti-CXCR3 neutralizing antibodies (figure 3A). Similarly, the migration of DOT cells toward SW620 (figure 3B) or HCT-116 cells (online supplemental figure S3A) was strongly impaired by AMG487 treatment, further supporting CXCR3 dependence. In line with this, while inhibition of individual ligands (αCXCL9, αCXCL10, or αCXCL11) from CRC cell supernatants had minimal effect, consistent with ligand redundancy, simultaneous neutralization of all three ligands markedly impaired DOT-cell migration (figure 3C). Moreover, CXCR3 inhibition partially reversed the decline in CXCR3 expression levels on DOT cells after incubation with tumor cells, indicating decreased receptor engagement (online supplemental figure S3B). These results demonstrate that the CXCR3 signaling axis is a critical mediator of DOT-cell migration in vitro.

To assess the impact of CXCR3 inhibition in vivo, we first employed a systemic therapeutic approach using the established CRC xenograft model. 3 weeks after tumor inoculation, mice received a single dose of 10⁷ DOT cells, followed by daily injections with AMG487 for 1 week (online supplemental figure S4A). While DOT cell levels in the blood and spleen remained unchanged, the number of tumor-infiltrating DOT cells was significantly reduced in AMG487-treated mice (online supplemental figure S4B-E). To rule out potential off-target effects of systemic treatment, we next adopted a targeted approach (figure 3D). DOT cells were cultured in vitro with AMG487 for 24 hours and injected into tumor-bearing mice the following day. 1 week later, mice were euthanized, and tumor infiltration was assessed. In alignment with our previous results, AMG487-treated animals exhibited reduced tumor infiltration by DOT cells compared with controls, while no significant differences were observed in blood or spleen (figure 3E–F). Of note, DOT-cell phenotype remained largely unaltered following treatment with AMG487 across all analyzed organs (online supplemental figure S4F-G). Taken together, these findings indicate that DOT-cell migration strongly depends on CXCR3 and is impaired upon inhibition of this signaling pathway.

### PTPN2/PTPN1 inhibition enhances CXCR3-mediated DOT-cell recruitment and CRC control

**Figure 4:**
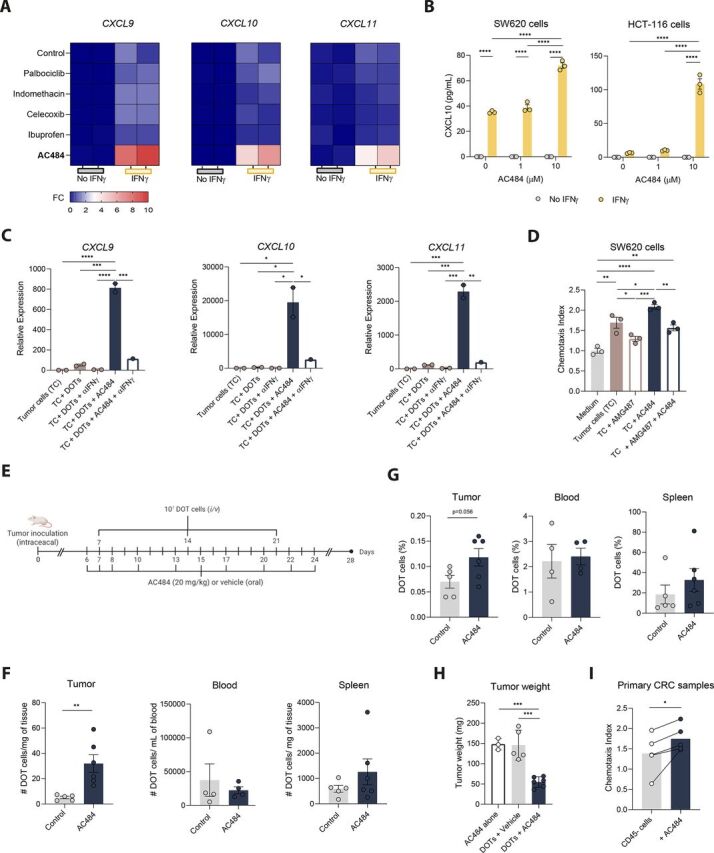
PTPN2/PTPN1 inhibition promotes CXCR3-mediated DOT-cell infiltration and CRC tumor control. (A) Heatmaps showing upregulation of CXCR3 ligands expression in SW620 cells following 24 hours exposure to different drugs, in the presence or absence of rIFNγ (100 ηg/mL). Data are presented as fold change relative to control (medium only) without rIFNγ. (B) Concentration of CXCL10 in the supernatants of SW620 or HCT-116 cells, after 3-day culture with varying concentrations of AC484 (0, 1 or 10 µM), with or without rIFNγ (100 ηg/mL), measured by ELISA. Representative experiment out of two independent experiments. Data are presented as mean±SEM and were analyzed by a two-way ANOVA with Tukey’s multiple-comparisons test. (C) Expression of *CXCL9*, *CXCL10,* and *CXCL11* by SW620 cells after 24 hours co-culture with freshly thawed DOT cells, in the presence or absence of neutralizing anti-IFNγ antibody (10 µg/mL) and/or AC484 (10 µM). Representative experiment with two technical replicates, presented as mean±SEM. (D) Chemotaxis index of DOT cells, treated or not with AMG487 (1 µM), in response to 8×10^5^ SW620 cells incubated overnight, with or without AC484 (10 µM). Medium without tumor cells served as the control. Data represented as mean±SEM from one representative experiment out of two independent experiments and analyzed by a two-way ANOVA with Tukey’s multiple-comparisons test. (E) Schematic representation of the in vivo experimental approach. Animals were treated with AC484 or vehicle by oral gavage daily for 3 days starting on day 6 after tumor inoculation, and then every 2 days from day 8 onward. In parallel, 10 million DOT cells were injected intravenously weekly into mice. (F) DOT cell numbers (human CD45^+^ cells) per mg of tissue or per mL of blood from control and AC484-treated animals. (G) Percentages of DOT cells (gated as human CD45^+^ cells among alive cells) in tumor, blood, and spleen from control and AC484-treated animals. (H) Tumor weights at day 28 in mice treated with DOTs or AC484, alone or in combination (n=5/6 animals per group). Data in G–H are presented as mean±SEM andn were analyzed using unpaired t-tests for normally distributed data and Mann-Whitney tests for non-normally distributed data. (I) Chemotaxis index of DOT cells in the presence of CD45^−^ cells isolated from primary CRC specimens and incubated overnight with rIFNγ (100 ηg/mL), with or without AC484 (10 µM). Lines connect paired data points from matched donors (n=5 donors). Data represent a pool of three independent experiments and were analyzed by a paired t-test. *p< 0.05, **p<0.01, ***p<0.001, ****p< 0.0001. ANOVA, analysis of variance; CRC, colorectal cancer; DOT, Delta One T; FC, fold change; IFNγ, interferon-gamma; i/v, intravenous.

Given the pivotal role of CXCR3 in DOT-cell migration, we hypothesized that enhancing the production of its ligands could promote DOT-cell infiltration and ultimately improve CRC tumor control. Building on previous studies, we selected candidate drugs that have been shown to induce the secretion of CXCR3 ligands, namely cyclooxygenase inhibitors (indomethacin, celecoxib, and ibuprofen),^17 18^ cyclin-dependent kinases CDK4/6 inhibitor (palbociclib),^19^ and protein tyrosine phosphatases PTPN2/PTPN1 inhibitors (ABBV-CLS-484, AC484).^8^ RT-qPCR analysis of SW620 cells revealed that AC484 was the drug that most markedly upregulated the expression of all three CXCR3 ligands upon IFNγ stimulation (figure 4A). Through PTPN2/1 inhibition, which dephosphorylates key components of the JAK/STAT pathway, AC484 de-represses downstream signaling, thereby activating IFNγ–mediated responses. To validate these findings at the protein level, SW620 and HCT-116 cells were treated with AC484 for 3 days, and supernatants were analyzed by ELISA. In alignment, increasing concentrations of AC484 led to elevated CXCL10 levels in both cell lines’ supernatants in an IFNγ-dependent manner (figure 4B), without affecting the viability of tumor cells (online supplemental figure S5A). Likewise, co-culture of tumor cells with AC484 in the presence of DOT cells confirmed that DOT-cell–derived IFNγ was sufficient to drive AC484-mediated upregulation of *CXCL9*, *CXCL10*, and *CXCL11* in tumor cells (figure 4C). Accordingly, chemotaxis assays revealed AC484-treated tumor cells markedly enhanced DOT-cell migration, an effect that was significantly reversed by the CXCR3 inhibitor AMG487, confirming that AC484 promotes DOT-cell trafficking through CXCR3 ligand engagement (figure 4D and online supplemental figure S5B). Consistent with this mechanism, CXCR3 surface levels on DOT cells (as a surrogate of engagement) were further reduced in the presence of AC484-treated tumor cells, which was reverted in the presence of AMG487 (online supplemental figure S5C).

Finally, to evaluate the therapeutic potential of AC484 in vivo, CRC-bearing mice were treated with AC484 by oral gavage daily for the first 3 days after tumor detection, and subsequently every other day for 3 weeks. 10^7^ DOT cells were administered intravenously weekly, starting on day 7 after tumor inoculation (figure 4E). After excluding a direct effect on tumor cells (online supplemental figure S5A), we examined whether the drug exerted cell-intrinsic effects on DOT cells. Exposure of DOT cells to 1–10 µM AC484 over 3 days did not interfere with viability and only slightly increased proliferation of DOT cells (online supplemental figure S5D-E). The cytotoxic profile of DOT cells was minimally changed by AC484, only displaying increased Granzyme B and decreased tumor necrosis factor (TNF) production (online supplemental figure S5F). Notably, AC484 treatment substantially increased both the number and percentage of tumor-infiltrating DOT cells, while no differences were observed in the blood or spleen (figure 4F and G). Characterization of infused DOT cells revealed that, besides lower NKG2D levels in tumors of AC484-treated mice, likely reflecting receptor internalization following ligand engagement, no major differences were observed in the phenotype of DOT cells between the two groups (online supplemental figure S5G-H). As a result of increased infiltration, AC484-treated animals, and only in combination with DOT cell infusion, exhibited a substantial reduction in tumor weight compared with controls, supporting the ability of this CXCR3-ligand de-repressor to enhance DOT-cell-mediated tumor control (figure 4H).

As a validation of these findings in a more clinically relevant context, we tested the effect of AC484 on DOT-cell migration toward primary CRC specimens. In alignment with our previous results, treatment of primary CD45-depleted CRC cells with AC484 led to a marked increase in DOT-cell migration (figure 4I), underscoring the therapeutic potential of leveraging this approach to enhance DOT-cell recruitment in patients with CRC.

In conclusion, this study identifies for the first time the key mechanisms driving DOT-cell migration toward CRC, highlighting CXCR3 as a central regulator of this process. This adds important mechanistic insight to previous studies demonstrating that other chemokine–chemokine receptor axes control γδ T-cell recruitment into different tumors in mice or humans, including CCR2-CCL2,^11^ CCR5-CCL4/5,^12^ CCR4-CCL17/22 and CCR8-CCL17.^13^

CXCR3 is broadly expressed on multiple immune cell subsets, including T lymphocytes, NK cells, and B cells, and has been recognized as a crucial mediator of immune cell infiltration across several solid tumors, including CRC.^7^ Although γδ T cells, particularly the Vδ1 subset, are known to infiltrate CRC tumors,^3 4^ and such infiltration has been associated with improved patient survival,^4^ the molecular mechanisms governing their recruitment have remained poorly defined. Here, we demonstrate that DOT cells, a Vδ1-enriched γδ T-cell product obtained from peripheral blood, acquire prominent CXCR3 expression during expansion that drives migration toward CRC cell lines and patient-derived specimens, as well as toward orthotopic xenografted CRC tumors in vivo. Our data further suggest that this mechanism may also rule endogenous Vδ1 T cells, highlighting the potential of actioning this axis to promote infiltration and CRC control from both endogenous and adoptive Vδ1 T cell-based immunotherapies.

The importance of CXCR3 signaling was further supported by inhibition studies using the selective antagonist AMG487.^16^ Although genetic depletion of CXCR3 could further strengthen these findings and should be performed in future studies, CXCR3 inhibition with AMG487 impaired DOT-cell migration in vitro and reduced tumor infiltration in vivo, consistent with its known effects on limiting lymphocyte trafficking into intestinal tumors.^20^ A comparable inhibitory effect was observed when all CXCR3 ligands were simultaneously neutralized to overcome functional redundancy.

Elevated levels of CXCL9 and CXCL10 have been positively associated with increased immune cell infiltration across multiple tumor types.^7^ In CRC specifically, a recent study showed that T cell populations reside within CXCL9-11-enriched niches in the TME.^21^ Here, we observed that overexpression of these ligands by the malignant CRC TME correlates with *TRDV1* expression, suggesting that CXCR3 may also contribute to the infiltration or retention of endogenous Vδ1 T cells. Of note, multiple cell types, including myeloid, cancer-associated fibroblasts and tumor cells have been implicated in the secretion of CXCR3 ligands in the TME.^7^ Thus, we propose that therapeutic modulation of the CXCR3–CXCL9/10/11 axis may offer a promising strategy to convert immune-cold tumors into immune-hot ones, enhancing not only the infiltration of adoptively transferred γδ T cells but also that of endogenous populations.

Immunotherapy of solid tumors is often limited by poor immune cell infiltration. ICB, which relies on the presence and activity of endogenous T cells, has shown limited efficacy in CRC, particularly in the more prevalent MMR-p subtype due to the scarcity of these cells within the TME.^22^ Similar limitations affect adoptive cell therapies, such as CAR-T cells, where insufficient capacity to infiltrate tumors remains a major barrier to therapeutic success. To address this caveat, chemokine receptors have been incorporated into CAR constructs to enhance tumor homing. Notably, CXCR3-engineered CAR-T cells have shown improved antitumor efficacy in murine models, including CRC.^23^ Here, we show that DOT-cell expansion naturally enhanced the expression of tumor-homing chemokine receptors, most notably CXCR3, expressed in virtually all DOT cells, and providing them with an inherent capacity to infiltrate CRC tumors without requiring additional engineering.

Among several compounds previously reported to induce CXCR3 ligand secretion, the PTPN2/1 inhibitor AC484 exhibited the most pronounced effect on chemokine production in CRC cell lines, which was validated in primary CRC samples. Consistent with previous reports showing that AC484 enhances cellular responsiveness to IFNγ,^8^ we observed that treatment of tumor cells with AC484 markedly increased the secretion of CXCR3 ligands, particularly in the presence of either recombinant or DOT cell–derived IFNγ. In vivo, AC484 has been shown to enhance antitumor immunity across several murine tumor models, including CRC, primarily by promoting endogenous CD8^+^ T-cell and NK-cell infiltration, likely in response to elevated levels of CXCR3 ligands.^8^ Given its strong therapeutic potential, AC484 (ABBV-CLS-484) is currently being evaluated in a clinical trial (NCT04777994) for patients with locally advanced or metastatic solid tumors. Similarly, PTPN2 deletion has been reported to augment tumor immunosurveillance by enhancing immune cell activation and function, as well as improving CAR-T cell homing (Wiede *et al*, 2020) and performance against solid tumors.^24 25^ In line with these findings, our results show that in vivo AC484 treatment enhances DOT-cell infiltration into CRC tumors and effectively limits tumor growth. Not only increased tumor-recruitment but also the increase in Granzyme B production by DOT cells, as described for CD8^+^ T cells in syngeneic models,^8^ could contribute to improved tumor killing.

Despite the physiological relevance of our orthotopic (intracecum) CRC xenograft model, it presents inherent limitations that should be acknowledged, most notably the absence of a human immune system, which prevents the evaluation of key immune components such as regulatory T cells or myeloid-derived suppressor cells that could influence tumor and treatment behavior. Future complementary work in immune-inclusive systems like organoid-based models would strengthen the translatability of our findings.

Altogether, our study identifies the CXCR3–CXCL9–11 axis as a dominant mechanism driving DOT-cell homing to CRC tumors and highlights PTPN2/PTPN1 inhibition as a promising strategy to increase the antitumor potential of DOT cells and other γδ T-cell-based immunotherapies against CRC.^1^

## Data Availability

Data are available upon reasonable request. Data supporting the findings of this study are available from the corresponding authors on reasonable request.
