# Supplementary material for: CXCR3 signaling promotes Delta One T cell recruitment and antitumor efficacy in colorectal cancer
Source: J Immunother Cancer. 2026 May 28;14(5):e014668. doi: 10.1136/jitc-2025-014668 (PMC13223943; doi:10.1136/jitc-2025-014668)
Supplement: Supplementary data [file jitc-14-5-s002.pdf]

Supplementary Table 1: Primer sequences used for RT-qPCR

Gene	Primer Sequence
**β-actin (Human)**	Forward: 5' CTGGCACCCAGCACAATG 3'<br><br>Reverse: 5' GCCGATCCACACGGAGTACT 3'
**CXCL9 (Human)**	Forward: 5'-CCAGTAGTGAGAAAGGGTCGC-3'<br><br>Reverse: 5'-AGGGCTTGGGGCAAATTGTT-3'
**CXCL10 (Human)**	Forward: 5'-GTGGCATTCAAGGAGTACCTC-3'<br><br>Reverse: 5'-TGATGGCCTTCGATTCTGGATT-3'
**CXCL11 (Human)**	Forward: 5'-GACGCTGTCTTTGCATAGGC-3'<br><br>Reverse: 5'-GGATTTAGGCATCGTTGTCCTTT-3'

Carreira M, *et al. J Immunother Cancer* 2026; 14:e014668. doi: 10.1136/jitc-2025-014668
