# Supplementary material for: CXCR3 signaling promotes Delta One T cell recruitment and antitumor efficacy in colorectal cancer
Source: J Immunother Cancer. 2026 May 28;14(5):e014668. doi: 10.1136/jitc-2025-014668 (PMC13223943; doi:10.1136/jitc-2025-014668)
Supplement: Supplementary data [file jitc-14-5-s003.pdf]

Supplementary Table 2: List of antibodies and dyes used for flow cytometry

Marker	Fluorochrome	Clone	Company	Catalogue Number
**CCR2**	BV421	K036C2	Biolegend	357209
**CCR5**	AF647	HEK/1/85a	Biolegend	313711
**CCR6**	BV711	G034E3	Biolegend	353435
**CD3**	BUV805	SK7	BD	612894
**CD4**	APC-Cy7	RPA-T4	Biolegend	300518
**CD8**	BV711	RPA-T8	Biolegend	301044
**CD11b**	BV421	M1/70	Biolegend	101235
**CD27**	BV605	O323	Biolegend	302830
**CD45**	AF700	HI30	Biolegend	304023/4
**CD45**	RB613	HI30	BD	758752
**CD45RA**	BV510	HI100	Biolegend	304142
**CD69**	RB744	FN50	BD	570501
**CXCR3**	PECy7	CEW33D	Invitrogen	25-1839-42
**CXCR3**	BUV563	1C6/CXCR3	BD	741406
**CXCR4**	PE	12G5	Biolegend	306505

Carreira M, et al. J Immunother Cancer 2026; 14:e014668. doi: 10.1136/jitc-2025-014668

**CXCR4**	PECy7	12G5	Biolegend	306513
**CXCR5**	APC-Cy7	J252D4	Biolegend	356925
**CXCR6**	BV650	13B 1E5	BD	743600
**DNAM1**	FITC	11A8	Biolegend	338304
**DNAM1**	BV711	11A8	Biolegend	338334
**Epcam**	APC	9C4	Biolegend	324207
**Granzyme B**	RY703	GB11	BD	571462
**IFNγ**	RY610	B27	BD	571143
**NKG2D**	BV421	1D11	Biolegend	320822
**NKG2D**	BV785	1D11	Biolegend	320830
**NKp30**	BUV737	P30-15	BD	749128
**PD-1**	BV480	EH12.1	BD	566112
**PD-1**	APC-Cy7	EH12.2H7	Biolegend	329921
**Perforin**	APC	B-D48	Biolegend	353312
**TCR Vδ1**	FITC	REA173	Miltenyi	130-118-362
**TCR Vδ1**	APC	REA173	Miltenyi	130-119-145
**TCR Vδ2**	PerCP-Cy5.5	B6	Biolegend	331423
**TCR Vδ2**	APC	123R3	Miltenyi	130-121-339

Carreira M, *et al. J Immunother Cancer* 2026; 14:e014668. doi: 10.1136/jitc-2025-014668

**TCR Vδ2**	APCVio770	REA771	Miltenyi	130-111-120
**TIGIT**	PECy7	A15153G	Biolegend	372717
**TIGIT**	PerCP-Cy5.5	A15153G	Biolegend	372735
**TIM3**	BV650	7D3	BD	565564
**TNFα**	BUV395	MAb11	BD	563996
**IgG1**	APC	MOPC-21	Biolegend	400122
**IgG1**	APC-Cy7	MOPC-21	Biolegend	400128
**IgG1**	BUV395	X40	BD	563547
**IgG1**	BUV737	X40	BD	612758
**IgG1**	BV421	MOPC-21	Biolegend	400157
**IgG1**	BV480	X40	BD	565652
**IgG1**	BV650	X40	BD	563231
**IgG1**	BV711	MOPC-21	Biolegend	400167
**IgG1**	BV785	MOPC-21	Biolegend	400169
**IgG1**	FITC	MOPC-21	Biolegend	400108
**IgG1**	PECy7	MOPC-21	Biolegend	400126
**IgG1**	RB744	X40	BD	570519
**IgG2a**	PE	MOPC-173	Biolegend	400213

**IgG2a**	PECy7	MOPC-173	Biolegend	400232
**IgG2A**	PerCP-Cy5.5	G155-178	BD	550927
**IgG2b**	APC	MPC-11	Biolegend	R2B6G3
**mouseCD45**	BV510	30-F11	Biolegend	102138
**Zombie Acqua**			BioLegend	423102
**Zombie Yellow**			Biolegend	77168
**Live/Dead NIR**			Invitrogen	L34976
**Cell Trace Yellow**			Invitrogen	C34567
**Cell Trace Violet**			Invitrogen	C34573

Carreira M, *et al. J Immunother Cancer* 2026; 14:e014668. doi: 10.1136/jitc-2025-014668
