# Supplementary material for: CXCR3 signaling promotes Delta One T cell recruitment and antitumor efficacy in colorectal cancer
Source: J Immunother Cancer. 2026 May 28;14(5):e014668. doi: 10.1136/jitc-2025-014668 (PMC13223943; doi:10.1136/jitc-2025-014668)
Supplement: Supplementary data [file jitc-14-5-s001.pdf]

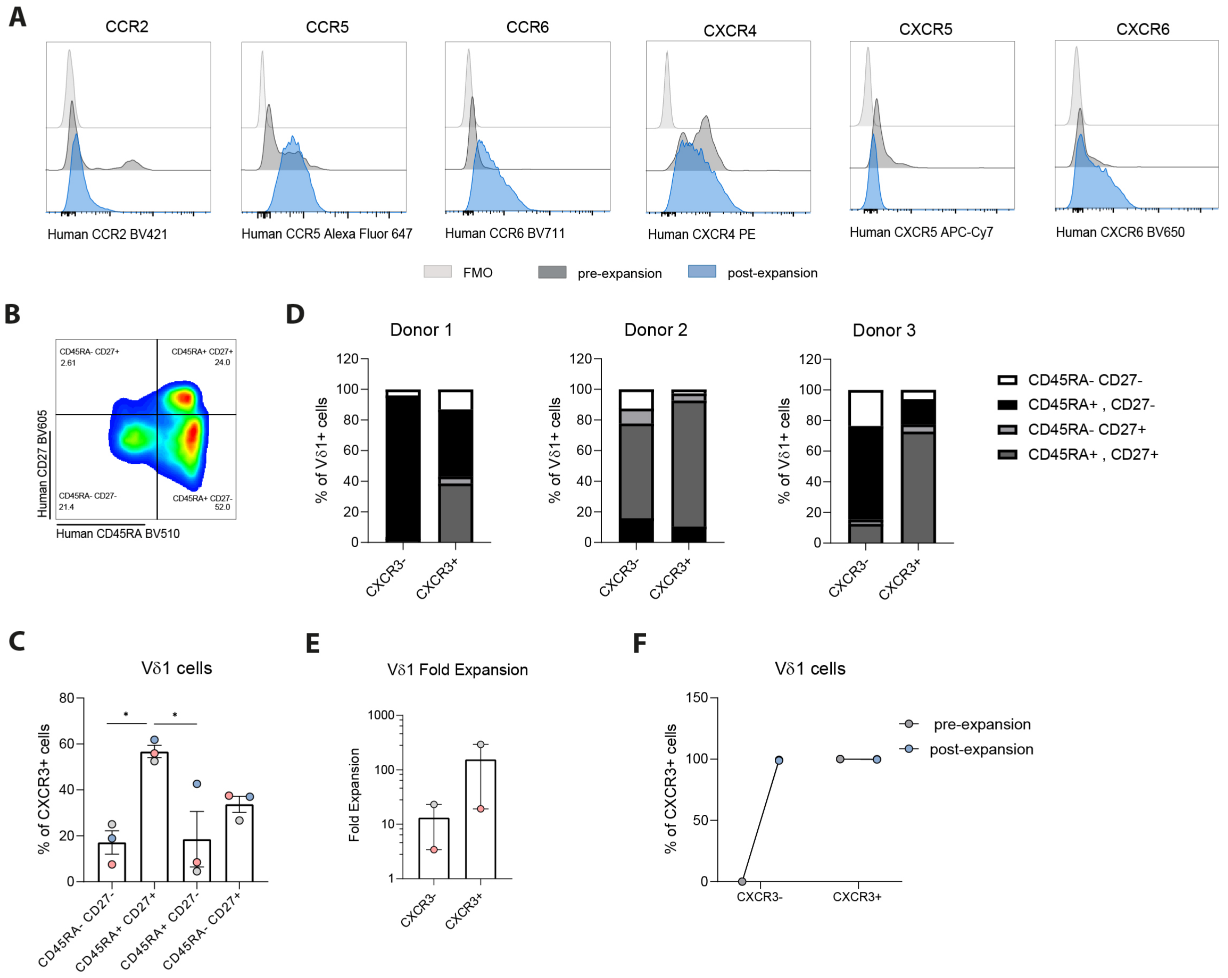

Supplementary Figure 1

Supplementary Figure 1. CXCR3 expression delineates a subset of naïve Vδ1^{+} T cells with enhanced expansion capacity. (A) Representative histograms showing the expression of various chemokine receptors by DOT cells before and after the expansion protocol. **(B)** Representative flow cytometry density plot illustrating the four Vδ1^{+} differentiation subsets defined by CD45RA and CD27 expression. **(C)** Frequency of CXCR3^{+} cells across Vδ1^{+} T-cell subsets defined by CD45RA and CD27 expression (gated on live cells). Each color represents an individual donor (n=3). Data represented as mean ± SEM and analyzed by one-way ANOVA with Holm–Sidak multiple comparison test. **(D)** Relative distribution (%) of the four Vδ1^{+} differentiation subsets (CD45RA^{-} CD27^{-}; CD45RA^{+} CD27^{-}; CD45RA^{-} CD27^{+}; CD45RA^{+} CD27^{+}) within the CXCR3^{-} and CXCR3^{+} populations in three independent DOT donors. **(E)** Fold expansion of DOT cells derived from CXCR3^{+} versus CXCR3^{-} starting populations. Each color depicts a different DOT donor (n=2). **(F)** Percentage of CXCR3^{+} cells before and after the DOT cell expansion protocol, starting from sorted CXCR3^{-} or CXCR3^{+} Vδ1^{+} cells (n=2 DOT donors).

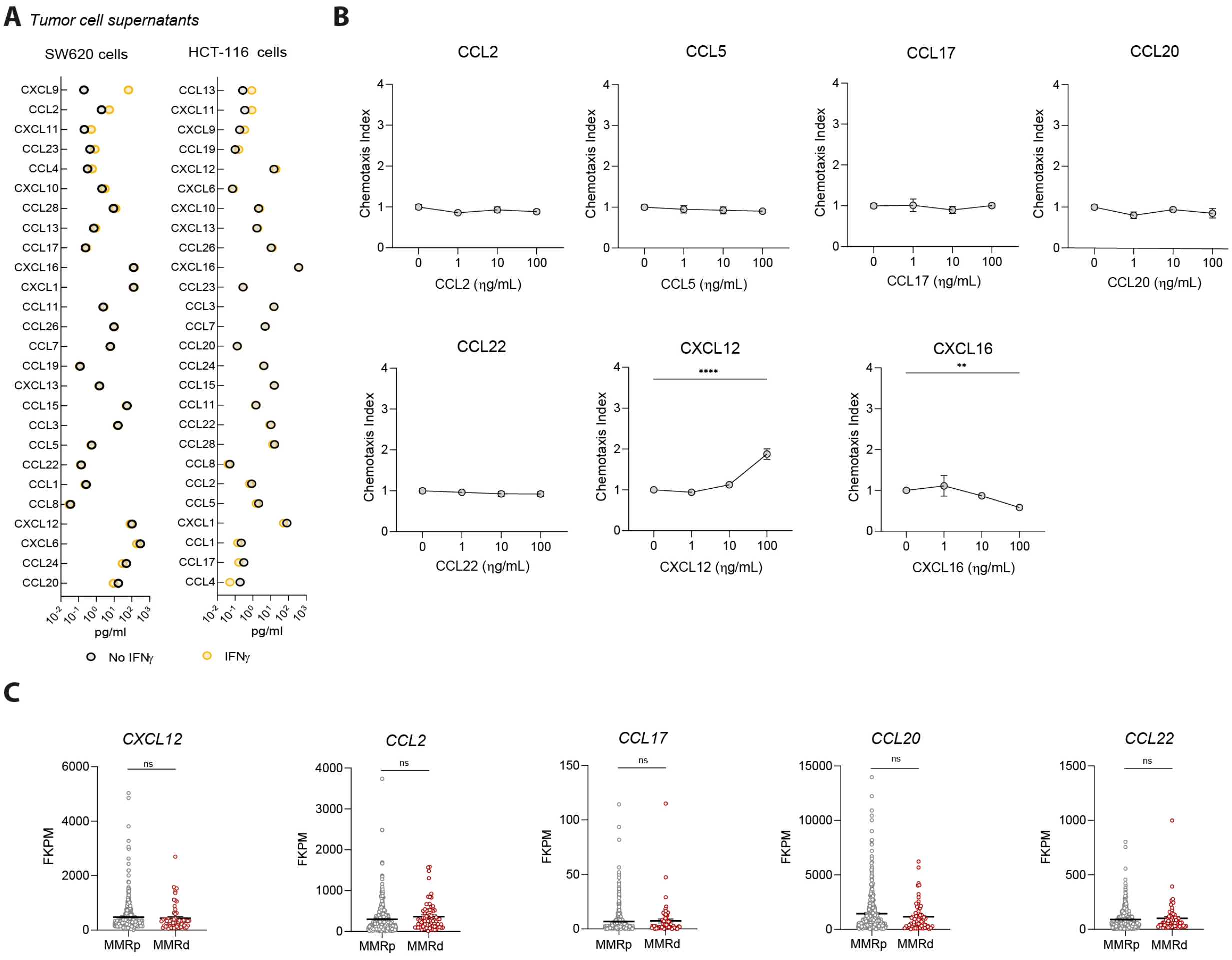

Supplementary Figure 2

Supplementary Figure 2. In vitro DOT cell-migration towards different chemokines and their expression in MMR-p and MMR-d CRC tumors. (A) Concentration levels of chemokines detected in SW620 and HCT-116 cell cultures in the presence or absence of IFNγ (10 ng/mL). **(B).** Chemotaxis index of DOT cells toward distinct chemokines (previously identified in panel **A**). Representative experiments with three technical replicates, represented as mean ± SEM. **(C).** *CXCL12, CCL2, CCL17, CCL20* and *CCL22* mRNA levels in primary samples from MMRp (n=468) and MMRd (n=70) CRC tumors, obtained from The Cancer Genome Atlas repository. Data presented as means ± SEM and analyzed by Mann-Whitney test. *p< 0.05, ****p< 0.0001, ns= not significant (p>0.05).

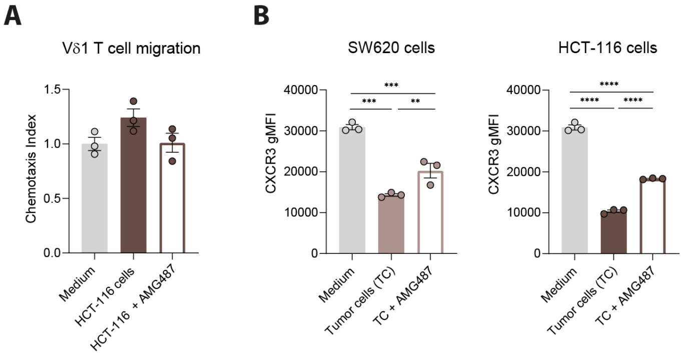

Supplementary Figure 3

Supplementary Figure 3. CXCR3 antagonist AMG487 impairs CXCR3-engagement and DOT-cell migration in vitro. (A) Migration index of DOT cells, treated or not with AMG487 (1 μM), in the presence of 8 × 10^5 HCT-116 cells incubated overnight with 100 ηg/mL of IFNγ. **(B)** Geometric mean fluorescence intensity of $CXCR3^+$ cells within $V\delta1^+$ T cells after DOT cell migration in response to SW620 or HCT-116 cells, with or without AMG487. Data represented as mean ± SEM from one representative experiment out of two independent experiments and analyzed by one-way ANOVA with Holm–Sidak multiple comparison test. **$p<0.01$, ***$p<0.001$, ****$p< 0.0001$

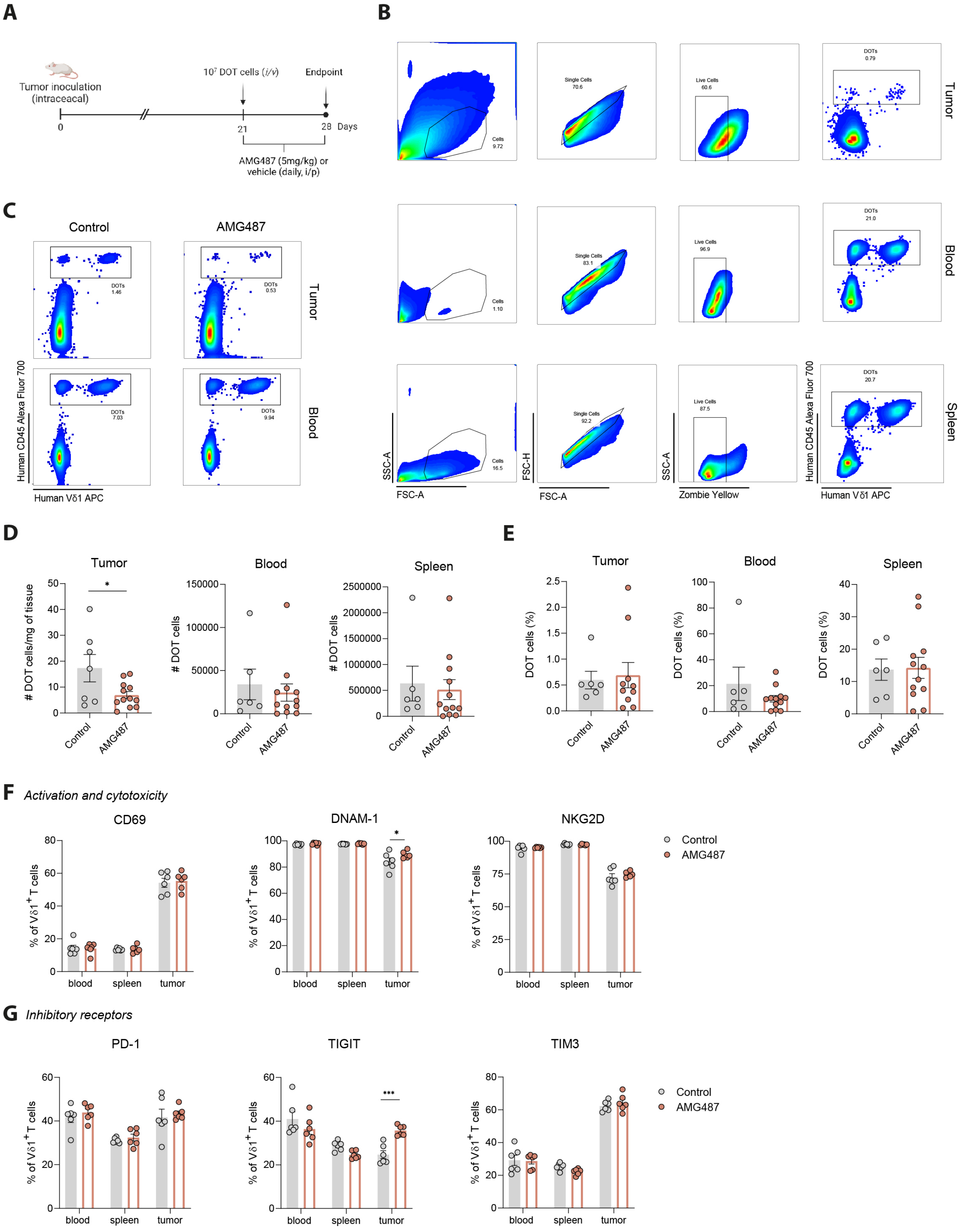

Supplementary Figure 4

Supplementary Figure 4. Systemic inhibition of CXCR3 efficiently limits DOT-cell infiltration into CRC tumors. **(A)** Schematic representation of the in vivo experimental approach to assess systemic inhibition of CXCR3. Three weeks after SW620 inoculation, mice were treated daily with AMG487 (5 mg/kg, i/p) or vehicle (DMSO + Corn Oil) for one week. **(B)** Flow cytometry density plots illustrating the gating strategy used to identify DOT cells across the analyzed organs (tumor, blood and spleen). **(C)** Representative flow cytometry density plots showing the percentages of DOT cells (gated on $CD45^+$ $V\delta1^+$ live cells) in tumor and blood from AMG487-treated and untreated animals. **(D)** Number of DOT cells ($hCD45^+$ cells) normalized per mg of tissue or per mL of blood. **(E)** Frequency of DOT cells in control and treated mice (n=7-12 mice per group). Data in **D-E** are represented as means ± SEM of a pool of two independent experiments and analyzed by unpaired t-tests for normally distributed data and Mann-Whitney tests for non-normally distributed data. Frequency of Vδ1+ cells expressing **(F)** activation and cytotoxicity receptors and **(G)** inhibitory receptors in DOT cells, either untreated or pre-treated *in vitro* with AMG487 (1 μM), derived from the tumor, blood, and spleen of the mice (n=6 mice per group). Data represented as mean ± SEM from one representative experiment out of two independent experiments and analyzed by two-way ANOVA with Sidak's multiple comparison test. *$p < 0.05$, ***$p<0.001$

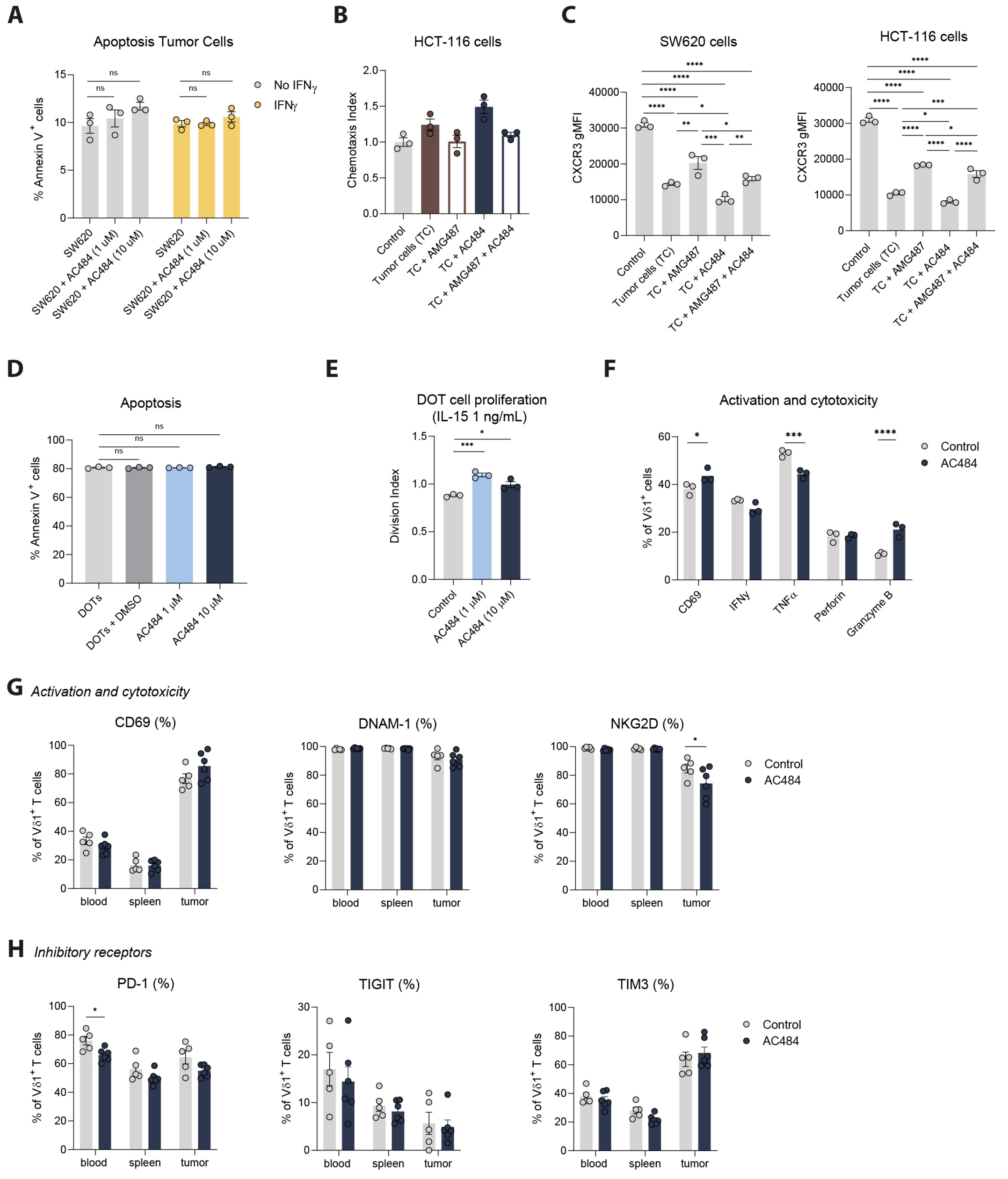

Supplementary Figure 5

Supplementary Figure 5. AC484 has minimal impact on DOT-cell phenotype or tumor cell viability. (A) Quantification of SW620 cell death after culture in the presence or absence of IFNγ, with or without AC484 (1 and 10 μM), assessed by annexin V staining. Data analyzed by two-way ANOVA with Tukey's multiple-comparisons test. **(B)** Chemotaxis index of DOT cells, treated or not with AMG487 (1 μM), in response to 8 × 10^5 HCT-116 cells incubated overnight, with or without AC484 (10 μM). Medium without tumor cells served as the control. Data from one representative experiment out of two independent experiments and analyzed by a two-way ANOVA with Tukey's multiple-comparisons test. **(C).** Geometric mean fluorescence intensity and percentage of CXCR3$^+$ cells within Vδ1$^+$ T cells after DOT cell migration in response to SW620 or HCT-116 cells, with or without AC484 and/or AMG487. One representative experiment out of two independent experiments and analyzed by a two-way ANOVA with Tukey's multiple-comparisons test. **(D)** Quantification of DOT-cell death following a 24h culture with DMSO or AC484 (1 and 10 μM), measured as the percentage of annexin V$^+$ cells. Data analyzed by one-way ANOVA with Dunnett's multiple comparisons test. **(E)** DOT-cell proliferation after 3 days of culture with AC484 (1 or 10 μM), assessed by division index (n=3 technical replicates). Data analyzed by one-way ANOVA with Dunnett's multiple comparisons test. **(F)** Expression of CD69, IFNγ, TNFα, Perforin, and Granzyme B in DOT cells (within Vδ1$^+$ live cells), in the presence or absence of AC484 (10 μM) for 3 days plus 3h stimulation with protein translocation inhibitors. Representative experiment with three experimental replicates, analyzed by two-way ANOVA with Šidák's multiple-comparisons test. Frequency of Vδ1$^+$ cells expressing **(G)** activation and cytotoxicity receptors and **(H)** inhibitory receptors in DOT cells

derived from the tumor, blood, and spleen from control and AC484-treated animals. One representative experiment out of two independent experiments analyzed by two-way ANOVA with Sidak's multiple comparison test. Data in panels **A-H** are represented as mean ± SEM. *$p < 0.05$, **$p<0.01$, ***$p<0.001$, ****$p< 0.0001$, ns= not significant ($p>0.05$).
